# Effect of intranasally administered insulin on cerebral blood flow and perfusion; a randomized experiment in young and older adults

**DOI:** 10.18632/aging.101192

**Published:** 2017-03-14

**Authors:** Abimbola A. Akintola, Anna M. van Opstal, Rudi G. Westendorp, Iris Postmus, Jeroen van der Grond, Diana van Heemst

**Affiliations:** ^1^ Department of Internal Medicine, section Gerontology and Geriatrics, Leiden University Medical Centre, Leiden, the Netherlands; ^2^ Department of Radiology, Leiden University Medical Centre, Leiden, the Netherlands; ^3^ Department of Public Health and Center for Healthy Aging, University of Copenhagen, Denmark; ^4^ Netherlands Consortium for Healthy Ageing, Leiden, the Netherlands

**Keywords:** intranasal insulin, cerebral blood flow, aging, phase contrast MR angiography, continuous arterial spin labelling (CASL), magnetic resonance imaging (MRI)

## Abstract

Insulin, a vasoactive modulator regulating peripheral and cerebral blood flow, has been consistently linked to aging and longevity. In this proof of principle study, using a randomized, double-blinded, placebo-controlled crossover design, we explored the effects of intranasally administered insulin (40IU) on cerebral blood flow (CBF) and perfusion in older (60-69 years, n=11) and younger (20-26 years, n=8) adults. Changes in CBF through the major cerebropetal arteries were assessed via phase contrast MR-angiography, and regional cortical tissue perfusion via pseudo-continuous arterial spin labelling. Total flow through the major cerebropetal arteries was unchanged in both young and old. In the older participants, intranasal insulin compared to placebo increased perfusion through the occipital gray matter (65.2±11.0 mL/100g/min vs 61.2±10.1 mL/100g/min, P=0.001), and in the thalamus (68.28±6.75 mL/100g/min versus 63.31±6.84 mL/100g/min, P=0.003). Thus, intranasal insulin improved tissue perfusion of the occipital cortical brain region and the thalamus in older adults.

## INTRODUCTION

Cerebral blood flow is pivotal for providing oxygen and nutrients to the brain to maintain brain function. The brain is a highly metabolically active organ but has only limited possibilities for energy storage. Thus, cerebral blood flow is important because the brain depends on constant and regulated blood supply to function properly. With aging, pathophysiological changes occur in large arteries causing decreased ability of large arteries to absorb and dampen pulsatile blood flow, eventually leading to pulsatile stress and arterial stiffness [1]. Transmission of increased pulsatile stress to the brain microvasculature is thought to lead to increased microvascular brain damage via altered dampening of pulsatile blood flow in cerebral arteries, and is considered a contributing factor for some cerebrovascular diseases [2].

Insulin influences all aspects of human physiology, including central regulation of energy homeostasis, cognitive functions and neuronal activity [3, 4]. The central regulation of energy homeostasis involves a complex neuronal network, which comprises the hypothalamus, the thalamus as well as cortical brain structures. Insulin and its signaling have been consistently linked to aging, lifespan and longevity [5, 6]. Aging is associated with reduced insulin action in peripheral tissues and possibly in the brain. Reduced insulin action in the aging brain may relate to age- associated cognitive defects and anomalies of metabolic homeostasis and an accelerated pace of aging [7-10]. Recent data suggest that insulin is also a vasoactive modulator that regulates peripheral and cerebral blood flow, possibly via a direct vasodilatory effect [11]. Reduced insulin action has been associated with vascular impairment possibly via impaired insulin- mediated augmentation of endothelium- dependent vasodilation, enhanced generation of reactive oxygen species and/ or excessive free fatty acids release from adipose tissue [12-16].

When administered in humans via the nasal route, insulin has been shown to distinctly alter brain functions, without being absorbed into the blood stream or having direct systemic effects on peripheral blood glucose or insulin levels [17]. Thus, the nasal route provides a safe and effective way of conveying insulin to the brain to specifically modulate brain insulin action and exert beneficial effects.

In this proof of principle, randomized, placebo-controlled trial, we investigated the effects of intranasal insulin administration on cerebral blood flow and perfusion in healthy older and young adults.

## RESULTS

Characteristics of the study subjects are shown in Table 1. The mean age of the older adults was 65.2 (range 60-69) years while the mean age of the younger adults was 22.3 years (range 20-26 years). The average BMI was similar in both groups of adults. For the younger adults, mean systolic blood pressure (SBP) was 125 mmHg (SD 9.3) and diastolic blood pressure (DBP) 74.5 mmHg (SD 7.2). For the older adults, mean SBP was 156 mmHg (SD 22.7) and mean DBP was 90.4 mmHg (SD 7.9). Fasted glucose, fasted insulin and HOMA index of insulin resistance were all within normal reference ranges for both groups of adults.

**Table 1:** Characteristics of study subjects

	Young		Older
**Demographics**	N = 8		N = 11
Age (years)	22.3 (1.8)		65.2 (3.3)
BMI (kg/m^2^)	23.6 (2.2)		24.1 (2.2)
Alcohol intake (units/week)	14.4 (2.2)		9.77 (1.7)
Systolic BP (mmHg)	125 (9.3)		156 (22.7)
Diastolic BP (mmHg)	74.5 (7.2)		90.4 (7.9)
Hypertension, n (%)	0 (0)		2 (18.2)
**Metabolic**			
Fasted glucose (mmol/L)	4.83 (0.24)		5.14 (0.57)
Fasted Insulin (pmol/L)*	5.3 (4.83, 6.58)		4.63 (2.9, 8.23)
HOMA-IR index*	1.21 (1.0, 1.49)		1.05 (0.65, 2.03)

Unless otherwise indicated, values are means (standard deviation). BMI: body mass index. BP: blood pressure. * Median (IQR).

### Plasma glucose and insulin trajectories

**Figure 1:**
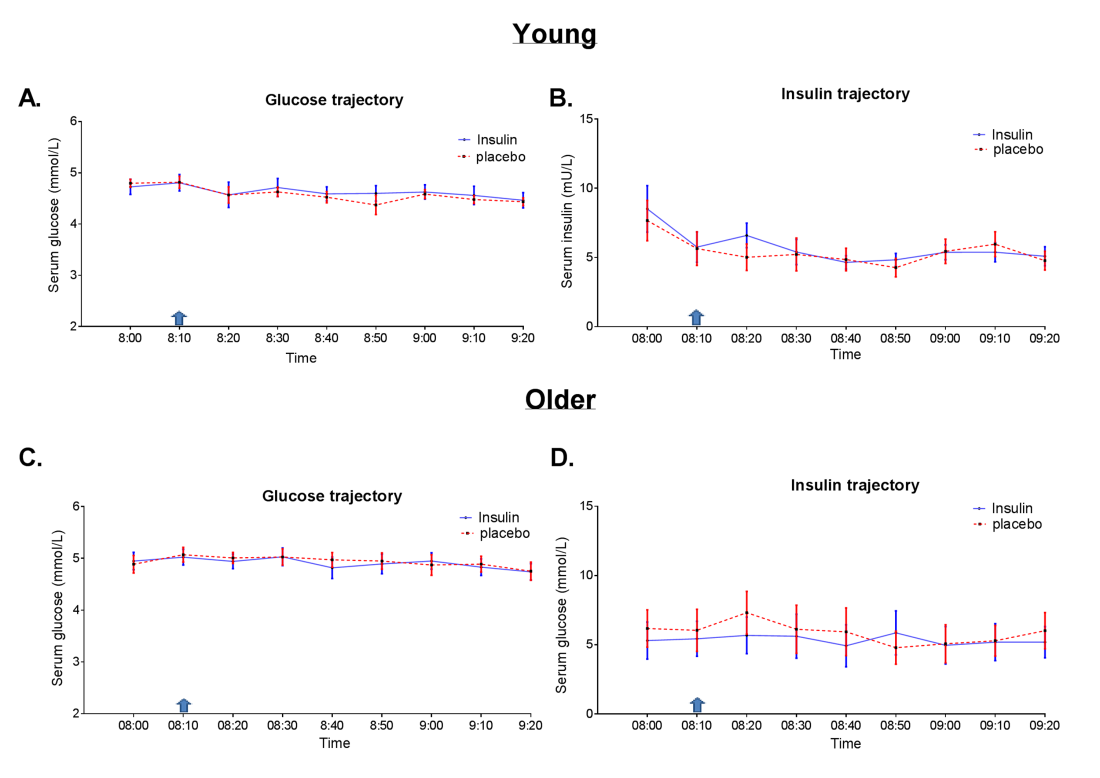
Glucose and insulin trajectories during the experimental period Concentrations (every 10 minutes) of glucose and insulin in blood serum over a 90- minute period, comprising measurements before and after intranasal application of insulin (40IU insulin Actrapid, blue line) or placebo (saline, red dotted line) using ViaNase nasal atomizer. Blue arrow indicates timing of intranasal administration of insulin or placebo. Data is presented as mean with standard error in (**A**) glucose trajectory in young and (**B**) insulin trajectory in young (**C**) glucose trajectory in older and (**D**) insulin trajectory in older adults.

We measured the peripheral (venous) glucose and insulin trajectories throughout an experimental period of 90 minutes, which comprised measurements before, during and after MRI scanning. As shown in Figure 1, the trajectories of blood glucose and insulin were similar and stable in insulin compared to placebo conditions throughout the experimental period, both in young and older adults. In the young adults, the mean (SD) glucose and insulin levels over the 90- minute period were 4.57 (0.3) mmol/L and 5.25 (2.7) mU/L respectively under placebo condition, while the mean (SD) glucose and insulin levels were 4.61 (0.5) mmol/L and 5.71 (2.6) mU/L respectively under insulin condition. Likewise, in the older adults, the mean (SD) glucose and insulin levels over the 90- minute period were 4.87 (0.5) mmol/L and 5.75 (3.4) mU/L respectively under placebo condition, while the mean (SD) glucose and insulin levels were 4.87 (0.5) mmol/L and 5.30 (3.6) mU/L respectively under insulin condition.

### Quantitative flow

The mean flow through the main cerebropetal arteries supplying the brain after intranasal application of insulin compared to placebo in both the older and younger adults is presented in Table 2. Intranasal application of insulin did not significantly change mean blood flow through the cerebropetal arteries in either older adults nor in the young. The mean difference in total quantitative flow under insulin minus placebo conditions in the younger adults was 0.35 ml/sec (SD 2.59), while that of the older adults was −0.24 ml/sec (SD 1.66).

**Table 2:** Quantitative flow through cerebropetal arteries

	Young			Older		
	Placebo Mean ± SD	Insulin Mean ± SD	p-value	Placebo Mean ± SD	Insulin Mean ± SD	p-value
**Arterial Flow**						
Basilar artery	3.36 ± 0.74	3.15 ± 0.42	0.376	2.91 ± 0.91	2.78 ± 0.63	0.313
Left carotid artery	4.44 ± 1.12	4.60 ± 0.78	0.745	4.48 ± 0.86	4.63 ± 0.69	0.537
Right carotid artery	4.24 ± 1.13	4.66 ± 0.81	0.263	4.64 ± 1.26	4.39 ± 0.75	0.532
Total flow	12.05 ± 2.73	12.40 ± 1.68	0.713	12.04 ± 2.26	11.8 ± 1.10	0.667

Cerebral blood flow (in ml/sec) ± Standard deviation (SD) through cerebropetal arteries in insulin conditions compared to placebo in young and older adults.

The variance (standard deviations) around the mean flow were consistently lower after insulin administration. A Levene's test verified the inequality of the variances in total quantitative flow after intranasal application of insulin compared to placebo when data from young and old adults were combined (P=0.047). Similar trends towards lower variances in cerebral blood flow after intranasal insulin application were observed in young and older adults.

### ASL perfusion

Perfusion through specific cortical regions of the gray matter is shown in Table 3. In addition to cortical regions, we also assessed perfusion in the thalamus and the hypothalamus. In younger adults, perfusion through the whole brain gray matter, frontal, parietal, temporal and occipital gray matter regions after intranasal administration of insulin was not significantly different when compared to placebo. In younger adults, intranasal administration of insulin did not change perfusion in the thalamus or in the hypothalamus.

**Table 3:** ASL perfusion through different brain regions

	Young			Older		
	Placebo Mean ± SD	Insulin Mean ± SD	p-value	Placebo Mean ± SD	Insulin Mean ± SD	p-value
**Brain regions**						
Whole brain	65.79 ± 11.69	67.53 ± 5.58	0.488	57.53 ± 8.0	59.0 ± 9.77	0.292
Frontal GM	74.93 ± 13.67	76.64 ± 5.52	0.603	63.11 ± 8.19	63.90 ± 10.72	0.653
Temporal GM	62.48 ± 10.82	64.39 ± 7.47	0.405	55.83 ± 7.22	55.74 ± 9.22	0.959
Parietal GM	77.2 ± 14.87	78.66 ± 7.04	0.653	66.35 ± 10.95	69.22 ± 11.51	0.034
Occipital GM	67.67 ± 13.21	68.27 ± 8.97	0.837	61.20 ± 10.11	65.15 ± 11.0	0.001*
Thalamus	65.43 ±10.27	65.84 ± 9.89	0.831	63.31 ± 6.84	68.28 ± 6.75	0.003*
Hypothalamus	43.97 ± 8.64	42.31 ± 6.43	0.603	38.31 ± 5.25	42.51 ± 6.43	0.156

Perfusion (ml/100g/min) ± Standard deviation (SD) through the whole brain, four major cortical gray matter regions, and subcortical regions in insulin conditions compared to placebo in young and older adults as measured by continuous arterial spin labelling. GM: gray matter. *= Remained significant after Bonferroni corrected threshold for significance has been set as 0.007.

**Figure 2:**
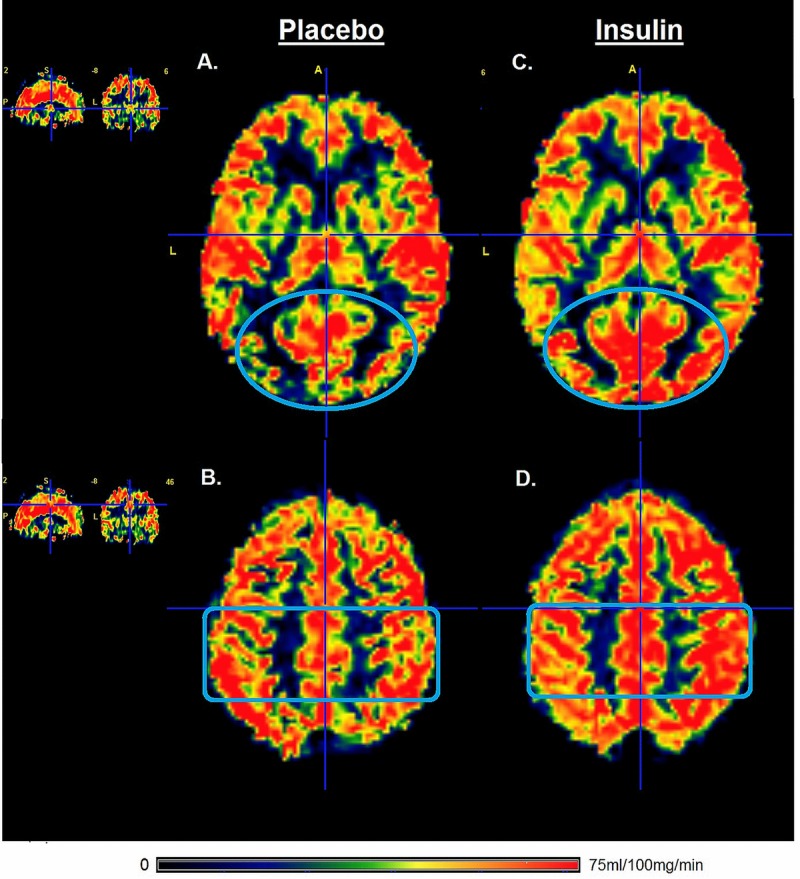
Gray matter perfusion maps after intranasal administration of placebo and insulin Left panel (**A** and **B**) represents the gray matter perfusion map after intranasal placebo administration and the right panel (**C** and **D**) the perfusion map after intranasal insulin administration for one representative older participant. The top row shows an increase in the gray matter perfusion of occipital lobe (illustrated by blue oval) after intranasal administration of insulin compared to placebo. The bottom row shows an increase in gray matter perfusion in the parietal lobe (illustrated by blue rectangle) after intranasal administration of insulin compared to placebo. Only the gray matter was included for calculation of perfusion after intranasal administration of insulin compared to placebo.

In older adults, intranasal administration of insulin significantly increased perfusion through the occipital gray matter with 6.5% when compared to the In older adults, intranasal administration of insulin significantly increased perfusion through the occipital gray matter with 6.5% when compared to the administration of placebo (Table 3, P=0.001). Perfusion through the parietal gray matter was 4.3% increased after intranasal administration of insulin (P=0.034). Perfusion in the frontal and the temporal gray matter regions was not significantly changed after intranasal administration of insulin (Table 3). In older adults, intranasal administration of insulin significantly increased perfusion in the thalamus (P=0.003), but no significant changes were found for perfusion in the hypothalamus. After application of Bonferroni corrected significance threshold of P<0.007, only the changes in occipital grey matter and the thalamus remained significant. The change in perfusion after intranasal application of insulin for one representative participant is visualized in the gray matter perfusion map shown in Figure 2.

In addition to the whole gray matter regions, we also investigated the perfusion through the right and left hemisphere of each region of interest (ROI) separately. The directions of observed effects in the left/right ROIs were comparable to those observed for the whole lobe ROIs. For example, in older participants perfusion in the right parietal region under placebo compared to insulin condition was 65.58 ± 11.70 compared to 68.31 ± 11.17 (p=0.061); and perfusion in the left parietal region under placebo compared to insulin condition was 67.12 ± 10.41compared to 70.12 ± 12.71 (p=0.221). Similarly, in older participants perfusion in the right occipital region under placebo compared to insulin condition was 60.92 ± 11.07 compared to 65.15 ± 10.97 (p=0.006); and perfusion in the left occipital region under placebo compared to insulin condition was 61.49 ± 9.35 compared to 65.13 ± 11.61 (p=0.005). Likewise, in older participants perfusion in the right thalamus under placebo compared to insulin condition was 63.79 ± 6.72 compared to 68.52 ± 6.14 (p=0.008); and perfusion in the left thalamus under placebo compared to insulin condition was 62.84 ± 7.11 compared to 68.03 ± 7.63 (p=0.005).

Analyses were repeated after exclusion of two older subjects that were hypertensive. The results did not materially change after exclusion of the two hypertensive older subjects.

**Figure 3:**
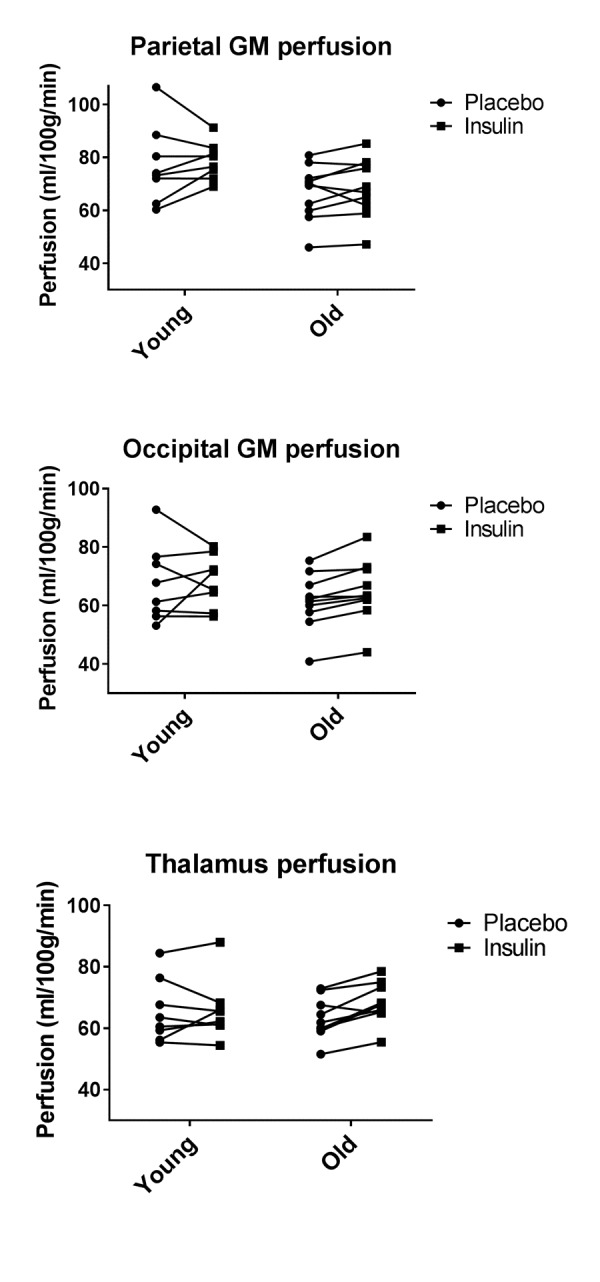
Paired individual gray matter perfusion measurements for placebo and insulin conditions Left panel represents the gray matter perfusion in the parietal lobe as paired data for placebo and intranasal insulin per individual subject in the young and the older group. The right panel represents the gray matter perfusion in the occipital lobe.

Figure 3 shows the paired gray matter perfusion in the parietal and occipital lobes and in the thalamus for every individual subject separately to illustrate individual differences between the placebo and insulin.

## DISCUSSION

This proof of principle study was aimed at exploring the effects of intranasal application of insulin on cerebral blood flow and perfusion in healthy young and older adults. We found no effect of intranasal insulin application on total quantitative flow, or on flow in the basilar, left or right carotid arteries, in either young nor older adults. However, region specific analysis indicated that intranasal insulin application increased perfusion in the occipital gray matter region and in the thalamus in older adults.

Due to the presence of direct pathways from the nasal cavity to the CNS, insulin can be delivered non- invasively rapidly to the CNS through the intranasal route without spilling over to the periphery. The intranasal route of insulin administration thus bypasses uptake into the bloodstream and so circumvents the risk of systemic hypoglycemia that is associated with peripheral administration. We found that blood glucose and insulin levels did not change after intranasal administration. This is in line with previous studies that show that plasma glucose concentrations remain unchanged after intranasal application of 40IU insulin [25, 26]. It further strengthens the fact that intranasal administration can safely be used for selectively increasing cerebral insulin levels.

One of the regions where insulin becomes readily available is the cerebral cortex, which is bathed by CSF, to which intranasally administered insulin has direct access [19]. There is evidence of bulk flow transport of peptides through extracellular pathways from the nasal cavity to the CNS, including cerebral cortex, gray matter and other parts of the CNS, via both olfactory and trigeminal pathways [27]. In the upper nasal cavity, olfactory neurons are exposed such that their axons project through the cribriform plate to the olfactory bulb. When inhaled, insulin accesses the CNS parenchyma either through the cribriform plate along the olfactory neurons or through perivascular channels associated with the olfactory or trigeminal systems [28]. In literature, different studies have used varying doses (10, 20, 40, 60 IU or 160IU) of insulin to elicit effects on CBF. In our study, we used 40IU of insulin. This dose was chosen based on the instrument that we used for insulin delivery. We used the ViaNase intranasal insulin device, which had been specifically designed to maximise drug transport to the central nervous system after delivery to the olfactory region. This device has been used extensively in literature, usually at a dose to of 20IU or 40IU of insulin, and has been shown to elicit beneficial effects on various endpoints, both in healthy persons and patients with Alzheimer's disease [25, 26].

Few studies have been performed to study the effect of intranasal application of insulin on regional blood flow in the brain. A recent study done in eight healthy young adults found no effect of 160IU intranasal insulin application on cerebral blood flow in the visual cortex, neither at baseline nor when cerebral blood flow was measured after specific tasks [20]. On the other hand, another study that was performed in a larger group of participants (25 lean, 10 overweight and 23 obese persons) of unspecified age found that 160IU intranasal insulin led to CBF decrease in the hypothalamus [29]. In our study, we found no effect of intranasal insulin application on flux in the major vessels, neither in young healthy men, nor in older men. It is possible that we missed a small effect of insulin on CBF because of the limited number of subjects in our study. Furthermore, since the positive finding in the aforementioned study was after administration of 160IU of insulin, it is possible that our dose of 40IU did not increase insulin concentration in the CNS sufficiently enough to effect changes in CBF.

While quantitative flow in the major arteries did not increase after intranasal application of insulin, we found that after intranasal insulin application, tissue perfusion was increased in the thalamus and in the occipital cortex (by 6.5%) in older adults. An increase of 4.3% was also found in parietal cortex in older adults, but which did not reach significance after strict correction for multiple comparisons. The occipital lobe is the visual processing center. It contains mainly the visual cortex and the ventral stream of vision that enables ability to focus on motor actions in response to outside stimuli. Next to the occipital lobe, the parietal lobe integrates sensory information among various modalities, including proprioception, mechanoreception, and visuospatial processing. The posterior parietal cortex, also referred to as the dorsal stream of vision, receives somatosensory and/or visual input that can be transmitted to motor signals. In a previous study done in 48 subjects with a mean age of 24 years, perfusion was increased in the insular cortex, an area closely related to the parietal cortex, after intranasal insulin administration [22]. Somewhat similar to the parietal cortex, the insular cortex also integrates information from other sensory modalities and contains topographically organized visceral sensory representation. We also observed that intranasal insulin application increased perfusion of the thalamus. The thalamus receives information from almost all sensory systems and relays the information to associated cortical areas. From literature, increased cerebral blood flow has been linked to vasodilatation around the active area due to increased energy demand [30]. Also, insulin has been shown to be a vasoactive modulator that regulates peripheral and cerebral blood flow possibly via a direct vasodilatory effect [11]. Taken together, our finding of increased perfusion in some brain areas would support the hypothesis that intranasal insulin application might restore energy demand and neuronal activity in these regions [7, 31].

This exploratory study is the first placebo-controlled, cross- over study in older and young adults showing that intranasally administered insulin increased regional perfusion in the occipital brain region and in the thalamus of older adults, as measured with a non- invasive ASL technique. Strength of our study is the cross-over design in which the participants were their own controls, thus minimizing the confounding effects of between- person differences. Moreover, both the young and older participants were included with strict in- and exclusion criteria. Thus, the characteristics of adults in both the young and older participant groups were homogenous, making them comparable within the group. On the other hand, this study has some limitations. It is limited by its sample size, especially of the younger age group, and the inclusion of males only. Thus, we cannot rule out that insulin has effect on many other brain areas, since several previous studies have demonstrated that insulin had beneficial effects on brain function including memory, cognition [32-34], and brain mediated functions such as postprandial thermogenesis and other metabolic profiles [31, 35-37]. Thus, other brain regions could have gone undetected because of the small sample size. We also cannot exclude the possibility that effects of intranasal administration of insulin may be sex specific. Indeed, sex-specific effects of intranasal administration of insulin have been observed for other endpoints, including reduction of body weight [36]. It is also a limitation that the clinical significance of the observed increases in CBF remains to be determined. Taken together, larger sufficiently powered studies are needed to delineate the exact effects of insulin on cerebral blood flow and perfusion in both the cortical brain regions and in subcortical structures in both males and females and in both young and older adults and to relate these to clinically significant outcomes.

## MATERIALS AND METHODS

### Ethics statement

This study titled “Maintaining health in old age through homeostasis: Switchbox Phase II” was approved by the Medical Ethical Committee of Leiden University Medical Center under protocol P13.164 and the Dutch competent authority (Centrale Commissie Mensengebonden Onderzoek (CCMO)) with protocol code number NL45043.058.13. The study is registered in the European Clinical Trials Database under number 2012-005650-29. All investigations have been conducted according to the principles expressed in the Declaration of Helsinki. All participants provided written informed consent after written and verbal description of the study was given.

### Trial design and participants

The design is a double-blinded, randomized, cross-over, placebo controlled trial in volunteers from the general population. This study was aimed at examining the effects of intranasal insulin on circulatory metabolic and endocrine parameters, as well as on resting and functional brain activities using MRI, in older and young adults. Fourteen elderly and nine young relatively healthy men (age criteria 60-85 years for older adults and 18-35 years for young adults), with a body mass index (BMI) between 20 kg/m2 and 27 kg/m^2^ were recruited from the general population. Exclusion criteria included fasting plasma glucose above 7 mmol/l, anemia (haemoglobin < 7.1 mmol/l), presence of anatomic deviations of the nose, any significant endocrine, neurological and cardiovascular diseases, or use of medication known to influence lipolysis, thyroid function, glucose metabolism, GH/IGF-1 secretion or any other hormonal axis. Furthermore, persons with a current smoking or alcohol addiction or history of substance abuse were excluded. Of the 23 participants that were contacted via telephone, three (older) participants were excluded after screening. The reasons for exclusion were uncontrolled high blood pressure (n=1) and renal insufficiency (n=2). Of these, all participants except for one (young) successfully completed the study. The reason for the drop-out was phobia for cannulas/ blood withdrawal.

### Randomization, masking and intervention

Randomization was carried out by the pharmaceutical trial coordinator, while the researchers were blinded to what the participants received. Concealment of treatment allocation was ensured by delivery of trial medication in a standard plain vial (same for all treatment groups) that had been prepared and re-labelled by the hospital pharmacy. De-blinding was done at the end of the study.

The study interventions consisted of intranasal application of 40 IU insulin (Actrapid; Novo Nordisk, Mainz, Germany) or placebo (Sterile saline), delivered using the ViaNase Electronic Atomizer (Kurve Technology inc.). The ViaNase device had been specifically designed to deliver drugs to the olfactory region to maximize drug transport to the central nervous system. The device released a metered insulin (40 IU) or saline dose directly into the subject's nostrils. A total volume of 0.4 mL (0.2 ml per nostril) was administered, which was inhaled by breathing evenly over a 2-minute period. In both right and left nostril, two doses each were delivered resulting in a total insulin dose of 40 IU. This method allowed administration of smaller particle sizes to increase drug deposition in the upper nasal cavity without transporting the drug to the lungs [18].

For the current study, results from two study visits, spaced apart by a week, on one of which they received insulin and on the other placebo, in a randomized manner were compared. All participants underwent exactly the same protocol during both study visits.

### Experimental protocol

**Figure 4:**
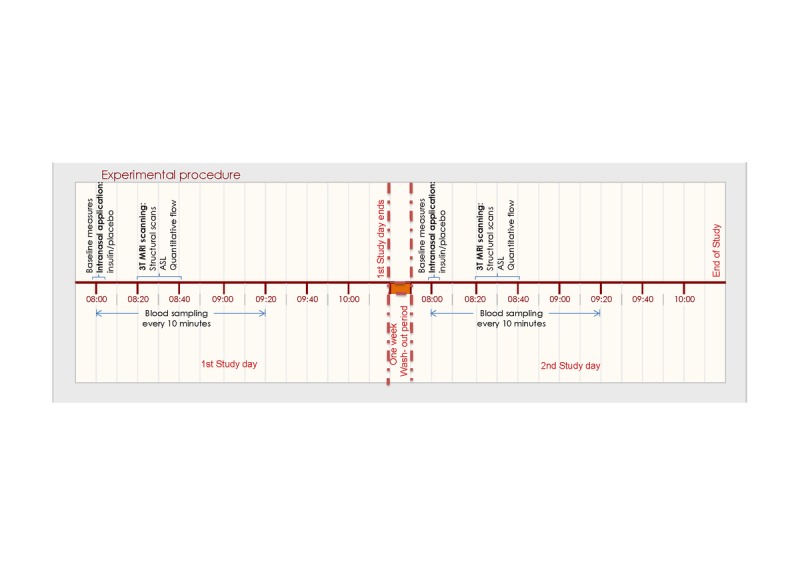
Flowchart of experimental procedures related to cerebral blood flow and perfusion measurements After an overnight fast and baseline measures, the study day started at 08.00 with blood sample withdrawal for baseline measures, after which subjects received either the intranasal insulin or intranasal placebo treatment. MRI scanning began 20 mins later, including survey/structural MRI scans, flow and perfusion scans.

In the morning after a 10-hour overnight fast, participants arrived at the MRI facilities at 08.00 in the morning. A catheter was placed in a vein of the forearm of the non-dominant hand. First, a baseline venous blood sample was collected in a serum-separator (SST)-tube. Thereafter, every 10 minutes, 4 ml of blood was collected into a SST-tube and 2 ml into K3-EDTA tube. Ten minutes after the first blood withdrawal, the trial medication (placebo or 40 IU of insulin) was administered intranasally, under the supervision of the experimenter, with a ViaNase Electronic Atomizer. Participants were asked if they noticed a specific smell or noticed something about the applied treatment. However, participants were not informed that the smell could be associated with the treatment and therefore would remain unaware of the applied intervention. We checked this by questioning the participants of the awareness of the treatment condition and this did not coincide with the actual applied treatment after de-blinding of the study. About 20 minutes after the intranasal application, participants were transferred into the MRI for the scanning procedure (Figure 4). About 30 min after the intranasal application, the CBF measurement was performed in the MRI scanner. This time interval was chosen because previous studies have shown that it takes 30 minutes for insulin to reach maximal concentrations in the CSF [19], and a similar time window has been used previously in other studies In the morning after a 10-hour overnight fast, participants arrived at the MRI facilities at 08.00 in the morning. A catheter was placed in a vein of the forearm of the non-dominant hand. First, a baseline venous blood sample was collected in a serum-separator (SST)-tube. Thereafter, every 10 minutes, 4 ml of blood was collected into a SST-tube and 2 ml into K3-EDTA tube. Ten minutes after the first blood withdrawal, the trial medication (placebo or 40 IU of insulin) was administered intranasally, under the supervision of the experimenter, with a ViaNase Electronic Atomizer. Participants were asked if they noticed a specific smell or noticed something about the applied treatment. However, participants were not informed that the smell could be associated with the treatment and therefore would remain unaware of the applied intervention. We checked this by questioning the participants of the awareness of the treatment condition and this did not coincide with the actual applied treatment after de-blinding of the study. About 20 minutes after the intranasal application, participants were transferred into the MRI for the scanning procedure (Figure 4). About 30 min after the intranasal application, the CBF measurement was performed in the MRI scanner. This time interval was chosen because previous studies have shown that it takes 30 minutes for insulin to reach maximal concentrations in the CSF [19], and a similar time window has been used previously in other studies where brain – related effects of intranasal insulin were studied [20-22].

### Anthropometrics

During the first study day, height, weight, percentage of body fat, and waist and hip circumference of participants were measured. Weight (in kilograms) was divided by the squared height (in meters) to calculate the BMI.

### Blood sampling and chemical analysis of blood samples

After each withdrawal, the K3-EDTA tubes were immediately placed on ice before centrifugation, whereas the serum tubes were kept at room temperature and centrifuged when the samples were clotted, usually between 30–60 minutes. Samples were centrifuged at 3520 RPM at 4 °C for 10 minutes. The EDTA plasma and serum samples were stored in two aliquots of 500 μl during the rest of the sampling at − 20 °C. After the sampling they were transferred to a − 80 °C freezer and stored until analysis.

All laboratory measurements were performed with fully automated equipment and diagnostics from Roche Diagnostics (Almere, The Netherlands). Glucose levels were measured using Hitachi Modular P800 from Roche (Almere, the Netherlands), with coefficient of variation (CV) for measurement less than 1%. Insulin levels were measured using the Immulite 2500 from DPC (Los Angeles, CA). CV was less than 6%.

### Brain MRI protocol

The perfusion measurement reported in the current study was part of a longer scanning protocol (48 minutes in young participants and 24 minutes in older participants) in which we aimed to successively assess several MRI endpoints. Scans were performed on a whole body magnetic resonance system with a 3 Tesla field strength (Philips Medical Systems, Best, the Netherlands). 3D T1-weighted images were acquired with the following imaging parameters: echo time (TE) 4.6ms, repetition time (TR) 9ms, Flip 8°, field of view (FOV) = 224 × 177 × 168mm, scan duration ~5minutes. The pseudo Continuous Arterial Spin Labeling (pCASL) with TE/TR/Flip: 14ms/4.0s/90°, FOV 240 x 133 x 240mm, matrix 80 x 80mm, slices 19, labeling duration of 1650 ms, post labeling delay ranging from 1525 ms for the most inferior slice to 2155 ms for the most superior slice scan, total scan duration 6 minutes. The M0-scan with TE/TR/flip: 14ms/6.0s/90, FOV 240x133x240, Matrix 80 80, slices 19, scan duration 30 seconds. The pCASL labelling slice was positioned perpendicular to the common carotid arteries right below the bifurcation point of common carotid into the internal and external carotid artery. The phase-contrast quantitative flow (QF) scan was planned using 2 localizer angiograms in the sagittal and coronal planes and acquired with the following parameters: TR = 11ms; TE 7.5ms; flip angle = 10°; slice thickness = 5mm; field of view 150x103mm; voxel size 1.17x1.17mm; velocity sensitivity = 200cm/s, 20 signal averages. Time allocated to measure perfusion was 6 minutes.

### Phase contrast MR Angiography

**Figure 5:**
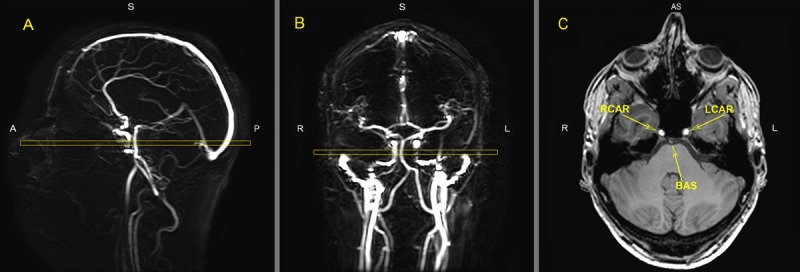
MR images of the vasculature measured with phase contrast MR angiography imaging for Quantitative Flow (QF) Typical placement of the QF phase-contrast slice (C) through the right internal carotid artery (RCAR), left internal carotid artery (LCAR) and the basilar artery (BAS) using the sagittal (A) and coronal (B) localizer angiograms. ROI's were drawn around the three arteries in slice C to measure flow.

Quantitative flow measurements were performed using ungated phase-contrast MRA, based on 2 localizer MR angiograms in the sagittal and coronal orientation as shown in Figure 5. A region of interest (ROI) was drawn around the vessel lumen on the magnitude images by an experienced rater using Philips Software on a PACS (Philips Medical Systems, Best, The Netherlands) workstation with ROI measurement tools. Flux was measured for basilar artery and both internal carotid arteries separately. Total flux was calculated for all three vessels together.

### pCASL quantitative analysis

MR images were analyzed using FMRIB Software Library (FSL) (Analysis Group, FMRIB, Oxford, UK). [23] The perfusion was measured in the partial volume corrected gray matter volume. pCASL scans were performed in a total time of 6 minutes. pCASL images were first corrected for slice timing, gradient nonlinearities and subject motion using in-house software and different tools of the FMRIB Software Library (FSL) version 5.0.6.

Files were smoothed and after subtraction a mean CBF over the entire 6- minute pCASL scan was calculated, yielding one average perfusion map for the 6- minute scan. This map was then translated to MNI152 space using FLIRT and FNIRT with the conversion matrix based on the 3DT1 scans. The perfusion was measured in the corrected gray matter volume. This corrected gray matter was segmented in four cortical areas based on the Harvard Oxford probabilistic cortical atlas threshold of 25 percent [24]. These ROI templates were subsequently used with each subject's gray matter mask to calculate the mean gray matter CBF in units of mL/100g/min for each anatomical region. Perfusion in the hypothalamus and thalamus was calculated in a similar way by measuring perfusion on the CBF maps in thalamic/hypothalamic ROI using Harvard oxford atlas ROI templates for these regions.

One participant was noticed to have extremely tortious blood vessels. For this participant, it was difficult to draw a consistent region of interest for phase contrast MR Angiography. For same reason, he also had strongly decreased ASL labelling efficiency. He was excluded from subsequent analyses.

### Statistical Analysis

Descriptive statistics were used to summarize the characteristics of both study groups. Homeostatic model assessment of insulin resistance (HOMA-IR) was calculated by the product of the fasting insulin level (mU/L) and the fasting glucose level (mmol/L) divided by 22.5.

The relation between intranasal insulin and cerebral blood flow was determined using paired t-tests, and presented as means with standard deviation with corresponding *p*-values. Homogeneity of variance assumption was tested using Levene's test. Correction for inter- individual and intra- individual differences in perfusion was done by calculating normalized gray matter volumes according to MNI standard space registration. For statistical analyses, Statistical Package for Social Sciences (SPSS) software for windows (version 20.0) was used. Statistical significance was set as P< 0.007, which is the corrected threshold for significance after Bonferroni correction for multiple comparisons.
